# Molecular mechanisms of the Guizhi decoction on osteoarthritis based on an integrated network pharmacology and RNA sequencing approach with experimental validation

**DOI:** 10.3389/fgene.2023.1079631

**Published:** 2023-01-25

**Authors:** Yan Chen, Yan Xue, Xuezong Wang, Ding Jiang, Qinguang Xu, Lin Wang, Yuxin Zheng, Ying Shi, Yuelong Cao

**Affiliations:** ^1^ Shi’s Center of Orthopedics and Traumatology, Shuguang Hospital Affiliated to Shanghai University of Traditional Chinese Medicine, Shanghai, China; ^2^ Shanghai Municipal Hospital of Traditional Chinese Medicine, Shanghai, China; ^3^ Shanghai Sunshine Rehabilitation Centre, Shanghai Yangzhi Rehabilitation Hospital, Shanghai, China

**Keywords:** Guizhi decoction, osteoarthritis, network pharmacology, RNA sequencing, TNF signaling pathway

## Abstract

**Background:** Our aim was to determine the potential pharmacological mechanisms of the Guizhi decoction (GZD) in the treatment of osteoarthritis (OA) through an integrated approach of network pharmacological analyses, RNA sequencing (RNA-seq), and experimental validation.

**Methods:** The quality control and identification of bioactive compounds of the GZD were carried out by using ultra-performance liquid chromatography (UPLC), and their OA-related genes were identified through overlapping traditional Chinese medicine systems pharmacology database (TCMSP), DrugBank and SEA Search Server databases, and GeneCards. The Gene Ontology (GO) and Kyoto Encyclopedia of Genes and Genomes (KEGG) pathway analysis were implemented after constructing the component–target network. RNA-seq was used to screen differentially expressed genes (DEGs) under intervention conditions with and without the GZD *in vitro*. The crossover signaling pathways between RNA-seq and network pharmacology were then analyzed. Accordingly, protein–protein interaction (PPI) networks, GO, and KEGG analysis were performed using the Cytoscape, STRING, or DAVID database. The OA rat model was established to further verify the pharmacological effects *in vivo*. Hematoxylin–eosin (H&E) and safranin O/fast green (S-O) staining were used to grade the histopathological features of the cartilage. We verified the mRNA and protein expressions of the key targets related to the TNF signaling pathways *in vivo* and *in vitro* by qPCR, Western blotting (WB), and immunofluorescence assay. In addition, we also detected inflammatory cytokines in the rat serum by Luminex liquid suspension chip, which included tumor necrosis factor-α (TNF-α), interleukin-6 (IL-6), and interleukin-1β (IL-1β).

**Results:** Eighteen compounds and 373 targets of the GZD were identified. A total of 2,356 OA-related genes were obtained from the GeneCards database. A total of three hub active ingredients of quercetin, kaempferol, and beta-sitosterol were determined, while 166 target genes associated with OA were finally overlapped. The RNA-seq analysis revealed 1,426 DEGs. In the KEGG intersection between network pharmacology and RNA-seq analysis, the closest screening relevant to GZD treatment was the TNF signaling pathway, of which TNF, IL-6, and IL-1β were classified as hub genes. In consistent, H&E and S-O staining of the rat model showed that GZD could attenuate cartilage degradation. When compared with the OA group *in vivo* and *in vitro*, the mRNA levels of TNF-α, IL-1β, IL-6, matrix metalloproteinase 3 (MMP3), and matrix metalloproteinase 9 (MMP9) were all downregulated in the GZD group (all *p* < 0.05). The expression levels of anabolic proteins (Col2α1 and SOX9) were all higher in the GZD group than in the OA group (*p* < 0.05), while the expression levels of the catabolic proteins (MMP9 and COX-2) and TNF-α in the GZD group were significantly lower than those in the OA group (*p* < 0.05). In addition, the expression levels of TNF, IL-6, and IL-1β were upregulated in the OA group, while the GZD group prevented such aberrations (*p* < 0.01).

**Conclusion:** The present study reveals that the mechanism of the GZD against OA may be related to the regulation of the TNF signaling pathway and inhibition of inflammatory response.

## 1 Introduction

Osteoarthritis (OA) is characterized by an imbalance between catabolism and anabolism of joint tissues due to damage to surfaces of joints caused by injury, chronic inflammation, or persistent mechanical stress that includes compression forces and tensile strains (Meulenbelt et al., 2007; Glyn-Jones et al., 2015; Pereira et al., 2015). The pathological condition of OA is related to cartilage destruction; however, there have been no effective drugs to reverse OA progression (Goldring, 2000; Aurich et al., 2005). Current therapeutic strategies against OA, which include acetaminophen and non-steroidal anti-inflammatory drugs, primarily focus on pain relief. These medications have been linked to side effects such as gastrointestinal bleeding, kidney dysfunction, and heart disease in clinical trials (Langman et al., 1994; McAlindon et al., 2014). It is therefore an urgent need and growing public health concern to explore new ideal therapies that are safe, of low cost, and long acting in treating OA.

Traditional Chinese medicine (TCM) enjoys a long history of preventing and treating OA. TCM has been utilized as a remedy for symptoms or syndromes of OA over thousands of years as recorded in numerous literature. According to modern research findings, the bioactive components of different kinds of TCM formulas have exerted beneficial effects on OA through various mechanisms such as inhibition of apoptosis, angiogenesis, antioxidation, and counter proliferation (Wang et al., 2014; Zhang et al., 2014; Wang et al., 2015). Bearing the longest history of these, the Guizhi decoction (GZD) has been a classical herbal formula recorded in the theory of Shang-Han since the Eastern Han, in treating pathogenesis of OA according to the TCM theory. It is composed of *Ramulus Cinnamomi* (Gui Zhi), *Radix Paeoniae Alba* (Bai Shao), *Zingiber Officinale Roscoe* (Sheng Jiang), *Radix Glycyrrhizae* (Gan Cao), and *Fructus Jujubae* (Da Zao) in a content ratio of 3:3:3:3:2.

Several single components in the GZD, such as ginger, isoliquiritigenin, and *Glycyrrhiza glabra*, have documented therapeutic activities against OA by anti-inflammatory activities, attenuation of osteoclast genesis, and anti-angiogenesis in a wide assortment of cells (Jia et al., 2017; Ji et al., 2018). However, the comprehensive composition of GZD and its multiple targets, as well as the underlying mechanism against OA, have not been fully elucidated.

To investigate the multi-target pharmacological mechanism of TCM formulas, methods of systems biology have become more efficient and productive than individual drug studies. Molecular genetics and proteomics have provided holistic approaches for studying TCM and herbal compounds in recent years. With the design of genomic innovations and framework science through computational natural instruments, network pharmacology has become an emerging discipline in drug development. High-throughput RNA-seq is a vigorous transcriptional screening innovation that is used to distinguish differentially expressed genes (DEGs) through examinations of various circumstances, under ordinary and illness states (Zhang et al., 2021). The combination of bioinformatics and transcriptomics has improved the accuracy of gene and gene-related pathway analyses.

Therefore, by aiming to reveal the mechanism of GZD in OA treatment, our study primarily intends to identify bioactive components and common targets and detect disease-related genes and main signaling pathways through an approach of integrated ultra-performance liquid chromatography (UPLC), network pharmacology, RNA-seq, and experimental validation.

## 2 Materials and methods

### 2.1 Preparation of GZD

All the herbs in GZD, that is, *Ramulus Cinnamomi* (150 g), *Radix Paeoniae Alba* (150 g), *Zingiber officinale Roscoe* (150 g), *Radix Glycyrrhizae* (150 g), and *Fructus Jujubae* (100 g), were obtained from Shuguang Hospital Affiliated to Shanghai University of Traditional Chinese Medicine. After soaking in 1 L of water for 0.5 h, the herbs were boiled for 1.5 h, and then the filtrates were combined, concentrated under reduced pressure, and freeze-dried. The GZD freeze-dried powder was stored at 4°C for use in *in vitro* and *in vivo* experiments.

### 2.2 Quality control and component identification of GZD by UPLC

The emperor herb (*Ramulus Cinnamomi*) in GZD was selected for the quality control component. First, the quality of the emperor herb was controlled by a mass spectrometer. Then, the Q Exactive™ Plus hybrid quadrupole-Orbitrap mass spectrometer (Thermo Fisher, United States) was used to analyze the components of the GZD. The samples were analyzed in an ACQUITY UPLC BEH C18 column (2.1 × 100 mm, 1.7 μm) (Waters, United States) at 45°C. For qualitative analysis, the mass spectrometer (MS) was operated in the full-scan mode from m/z 80 to 1,200 and, for a quantitative analysis, SIM mode was applied.

### 2.3 Screening of potential targets in components and OA-related targets

The potential target information was obtained by searching the traditional Chinese medicine system pharmacology database (Ru et al., 2014) (TCMSP, https://tcmsp-e.com/), DrugBank (Law et al., 2014) (https://go.drugbank.com/drugs/), and SEA Search Server (https://sea.bkslab.org/). We employed oral bioavailability (OB) ≥ 30% and drug likeness (DL) ≥ 0.18 to detect the potential bioactive compounds, represented as the key contributors that influence bioactivities (Cui et al., 2020). We also implemented an electronic search in GeneCards (https://www. genecards.org) (Stelzer et al., 2016), using the string “knee osteoarthritis” to get OA-related targets.

### 2.4 Bioinformatics analysis

The Gene Ontology (GO) database (http://geneontology.org/), which included terms for biological processes, cell components, and molecular functions, was used to identify possible biological mechanisms using high-throughput genomic or transcriptome data. The Kyoto Encyclopedia of Genes and Genomes (KEGG) database (https://www.kegg.jp/) was used as a knowledge foundation for determining putative targets’ systematic functions and biological significance (Ashburner et al., 2000). Furthermore, the GO functional annotation and KEGG pathway analysis were performed using the DAVID database.

### 2.5 Protein–protein interaction analysis

The protein–protein interaction (PPI) analysis network was accomplished in the STRING database (https://string-db.org/), and the background organism was selected to *Homo sapiens*, while the other settings were set by default. The Cytoscape 3.6.0 program was employed for network visualization.

### 2.6 Chondrocyte culture and RNA-seq

We isolated chondrocytes from cartilage explants obtained from newborn mice. All experiments were performed using confluent P0 or P1 cells in the presence of 10% fetal bovine serum. The cells were divided into three groups: the normal (Control) group, IL-1β–induced (OA) group, and GZD freeze-dried powder intervention (GZD) group. This experiment was reviewed by the Welfare and Ethics Committee of Experimental Animals of the Shanghai University of Traditional Chinese Medicine (Ethics No: PZSHUTCM200710008). An aggregate sum of 1.5 μg RNA per sample was isolated from IL-1β–induced chondrocytes subjected to the GZD freeze-dried powder of 0 and 50 μg/mL concentrations for utilizing TRIzol (Invitrogen) as indicated by the guidelines. There were three biological replicates in each group. Sequencing libraries were generated using NEBNext^®^ Ultra^TM^ RNA Library Prep Kit for Illumina^®^ (NEB, United States) following the manufacturer’s recommendations, and index codes were added to attribute sequences to each sample. The DEGs were identified using the Cuffdiff software (a part of the Cufflinks software). The thresholds of the DEGs were set as fold-change (FC) log |FC| ≥ 1.0 and *p*-value <0.05, with a fragments per kilobase million (FPKM) value ≥0.1, and at least one criterion was expected to be satisfied. Differentially expressed mRNA clustering was performed using the FPKM values with the heatmap function of the R package.

### 2.7 Modeling and intervention of OA rats

A total of 30, five-week-old male Sprague–Dawley rats were provided by the Shanghai SIPPR-Bk Lab Animal Co., Ltd. This experiment was reviewed by the Welfare and Ethics Committee of Experimental Animals of the Shanghai University of Traditional Chinese Medicine (Ethics No: PZSHUTCM190322003).

After 1 week of acclimation, the rats were divided into three groups: a sham group (*n* = 10), an OA group (*n* = 10), and a GZD group (*n* = 10). The rats in OA and GZD groups all underwent improved Hulth's surgery to establish the OA rat model. First, the right medial knee joint approach of the rats was removed and the medial collateral ligament was cut. Then, the medial meniscus was removed and the anterior cruciate ligament (ACL) was severed (Hulth et al., 1970). Immediately after the surgery, the rats in the GZD group received a dose of GZD of 0.97 g kg^−1^·day^−1^. Simultaneously, the mice in the OA group received an equal amount of saline. All the rats were sacrificed using an overdose of pentobarbital at 4 weeks after the intervention. The cartilage tissues, serum, and right knee joints of the rats were collected.

### 2.8 Histopathological analysis

The knee joints were fixed for 24 h in 4% (vol/vol) neutral paraformaldehyde solution and then decalcified for 1 month in decalcifying fluid. The joint slices (4 μm) were cut and stained with safranin O/fast green (S-O) or hematoxylin and eosin (H&E) after dehydration and embedding them in paraffin. The histopathological analyses were performed by a person who was unaware of the experimental group, and the Mankin score was used to evaluate the different groups (Mankin et al., 1971).

### 2.9 qPCR

TRIzol (Cat. No. 15596–026, Invitrogen) was used to extract RNA from cartilage tissues and chondrocytes, followed by cDNA production with the RT reagent Kit (RR036Q, Takara, Japan). All qPCR studies were carried out by using the SYBR Green qPCR Master Mix (B21203, Bimake, China). The target gene expression was analyzed *via* the ΔΔCt method. The primers used for this assay, such as the tumor necrosis factor-α (TNF-α), interleukin-6 (IL-6), interleukin-1β (IL-1β), matrix metalloproteinase 3 (MMP3), matrix metalloproteinase 9 (MMP9), and glyceraldehyde-3-phosphate dehydrogenase (GAPDH), are listed in Table 1.

**TABLE 1:** Primers of the qPCR gene.

Gene	Forward primer	Reverse primer
TNF-α	5′-CTG​CAA​AGG​GAG​AGT​GGT​CA-3′	5′-TTG​CAC​CTC​AGG​GAA​GAA​TCT​G-3′
IL-1β	5′-TGA​TGT​TCC​CAT​TAG​ACA​GC-3′	5′-GAG​GTG​CTG​ATG​TAC​CAG​TT -3′
IL-6	5′-GAC​AGC​CAC​TCA​CCT​CTT​CA-3′	5′-TTC​ACC​AGG​CAA​GTC​TCC​TC-3′
MMP3	5′-TCG​GTG​GCT​TCA​GTA​CCT​TT-3′	5′-CTGGAGAA TGTGAGTGGGGT-3′
MMP13	5′-TGT​GCG​ACC​ACA​TCG​AAC​TT-3′	5′-ATA​CAG​CGG​GTA​CAT​GAG​CG-3′
GAPDH	5′- TTC​AAC​GGC​ACA​GTC​AAG​G-3′	5′-CTC​AGC​ACC​AGC​ATC​ACC-3′

### 2.10 Western blotting

After lysing the cartilage and chondrocytes in RIPA buffer containing 10% PMSF, the protein levels in the supernatants were determined using the BCA Protein Assay Kit (Cat. No 23227, Pierce, United States). Proteins were separated using 10% SDS-PAGE and were transferred to PVDF membranes, where they were treated with the appropriate primary antibodies and secondary antibodies GAPDH (#2118S, CST, United States), cyclooxygenase-2 (COX-2) (A5523, Bimake, China), MMP9 (#13667, CST, United States), collagen type II alpha-1 chain (Col2α1) (#34712, Abcam, United States), SRY-box transcription factor 9 (SOX9) (#185966, Abcam, United States), and TNF-α (#11948, CST, United States). In order to analyze the data and compare the groups, two phases of normalization were required. The ImageJ software was used to objectively assess the protein band grayscale values and adjust them to GAPDH or phosphorylated proteins to their total proteins.

### 2.11 Serum cytokine detection

Animal serum samples were collected from the abdominal aorta. The Luminex liquid suspension chip was used to detect the serum cytokines TNF-α, IL-6, and IL-1β. The principle followed was as per the Bio-Plex Pro™ Rat Cytokine Assay (Bio-Rad, United States) manual. A log regression standard curve was used to calculate the cytokine concentrations.

### 2.12 Immunofluorescence Assay

The cells were fixed in 4% PFA for 10 min at room temperature, permeabilized for 20 min with 0.1% Triton X-100, and blocked for 10 min (Beyotime QuickBlock^TM^ kit). The cells were treated with Col21 overnight at 4°C, washed thrice with PBST, and stained in the dark for 30 min with a fluorescent secondary antibody (SA00007-2, Proteintech, United States). Finally, the nucleus was labeled for 5 min with 10 mg/mL DAPI before the cells were examined under the fluorescence microscope (Olympus IX73, Tokyo, Japan). The ImageJ software was used to compute the mean fluorescence intensity.

## 3 Results

### 3.1 Ingredient analyses of GZD

**FIGURE 1:**
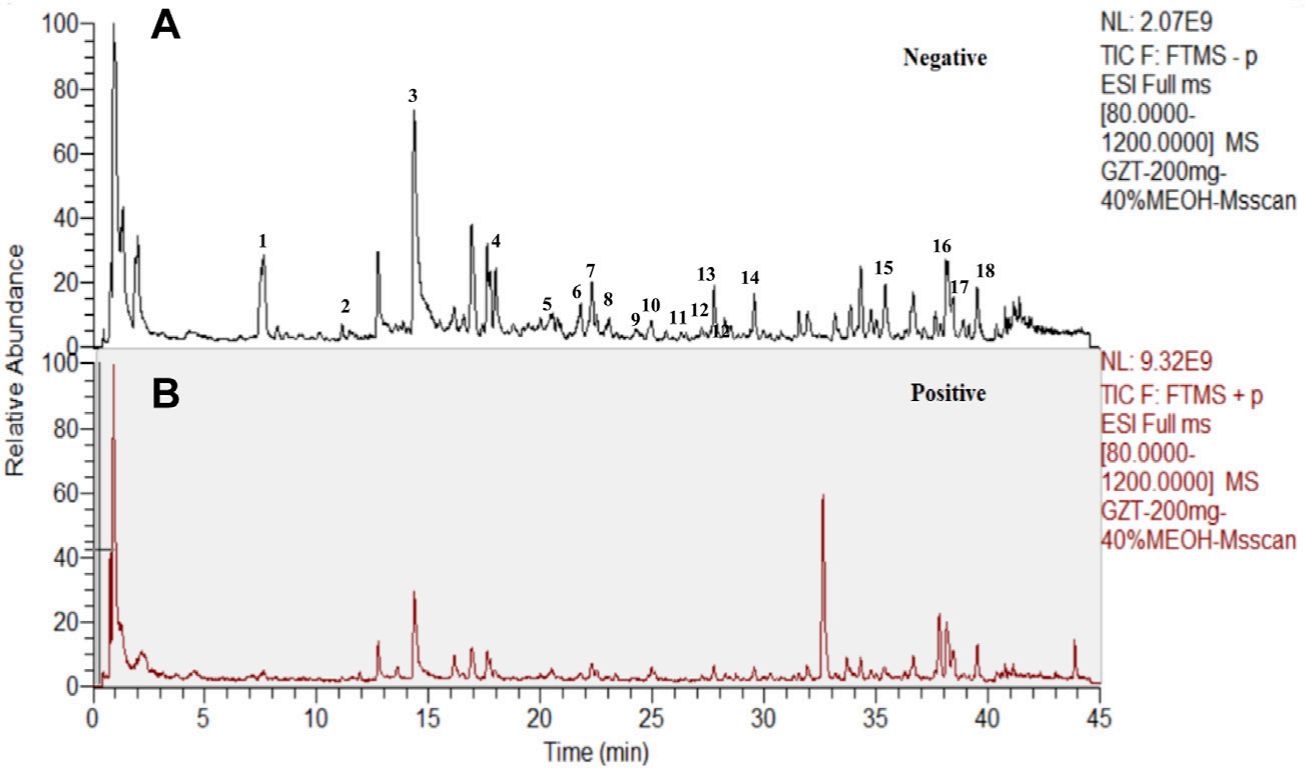
Eighteen compounds of GZD by UPLC [**(A)** negative, **(B)** positive ion mode]: 1. (+)-catechin, 2. paeoniflorigenone, 3. paeoniflorin, 4. pratensein, 5. lactiflorin, 6. liquiritigenin, 7. glycyroside, 8. licochalcone B, 9. quercetin, 10. licorice glycoside E, 11. echinatin, 12. kaempferol, 13. benzoylpaeoniflorin, 14. formononetin, 15. licoisoflavone A, 16. licochalcone A, 17. licoisoflavone B, and 18. beta-sitosterol.

The workflow of the present study is summarized in Supplementary Figure S1. We employed cinnamyl alcohol in the emperor herb (*Ramulus Cinnamomi*) as the significant factor in quality control (Supplementary Figure S2). The results showed that the peaks of cinnamyl alcohol appeared at 15.287 ± 0.003 min, which is consistent with the timing of GZD. The analysis of the ingredients of GZD was performed by using UPLC. Eighteen different molecular mass components (Table 2) were filtered by OB ≥ 30% and DL ≥ 0.18 in the analyzed GZD (Figure 1, Supplementary Table S1).

**TABLE 2:** Information of the 18 compounds in GZD.

Peak	RT/min	Measured mass/Da	Molecular formula	Identification
1	14.32	525.1614	C_23_H_28_O_11_	Paeoniflorin
2	35.41	353.1032	C_20_H_18_O_6_	Licoisoflavone A
3	21.8	255.0662	C_15_H_12_O_4_	Liquiritigenin
4	12.75	319.1173	C_17_H_18_O_6_	Paeoniflorigenone
5	27.74	629.1882	C_30_H_32_O_12_	Benzoylpaeoniflorin
6	20.75	507.1510	C_23_H_26_O_10_	Lactiflorin
7	22.92	285.0770	C_16_H_14_O_5_	Licochalcone B
8	38.28	351.0876	C_20_H_16_O_6_	Licoisoflavone B
9	29.91	267.0664	C_16_H_12_O_4_	Formononetin
10	26.48	269.0820	C_16_H_14_O_4_	Echinatin
11	24.23	695.1991	C_35_H_36_O_15_	Licorice-glycoside E
12	38.03	337.1450	C_21_H_22_O_4_	Licochalcone A
13	22.52	563.1757	C_27_H_30_O_13_	Glycyroside
14	8.62	289.0721	C_15_H_14_O_6_	(+)-Catechin
15	24.18	303.0498	C_15_H_10_O_7_	Quercetin
16	27.33	287.0549	C_15_H_10_O_6_	Kaempferol
17	19.43	301.0704	C_16_H_12_O_6_	Pratensein
18	39.5	821.3977	C_29_H_50_O	Beta-sitosterol

### 3.2 Target screening

We obtained 18 compounds from the UPLC analysis and collected the targets of these compounds through target fishing based on TCMSP, DrugBank, and SEA Search Server databases.

**FIGURE 2:**
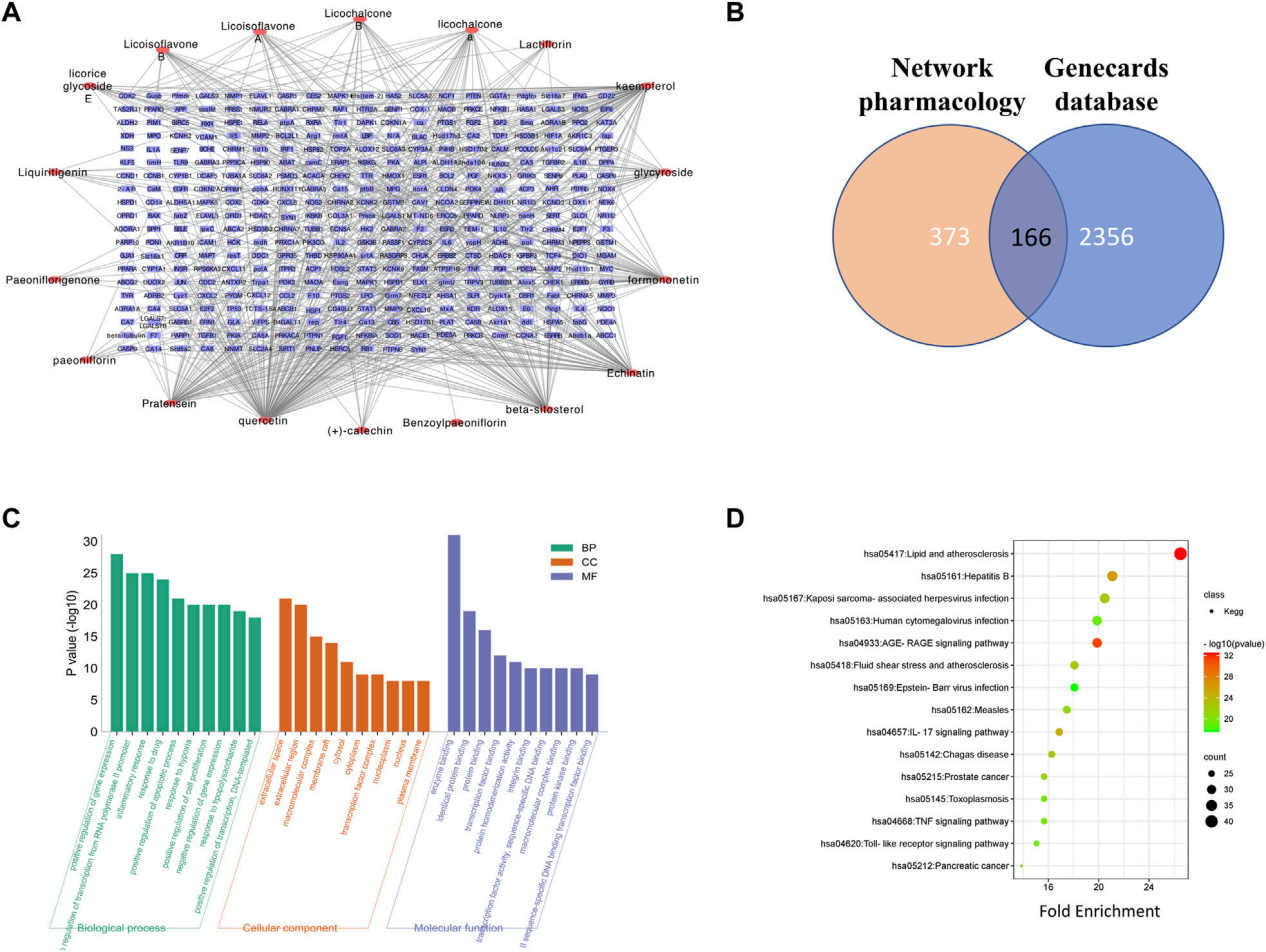
Network pharmacology analysis of GZD. **(A)** Compound–target network of GZD. **(B)** GO of the candidate targets of GZD against OA; the top 30 GO functional categories with a *p*-adjusted value <0.05 are selected. **(C)** KEGG pathway enrichment of candidate targets of GZD against OA. **(D)** Pathways that had significant changes of *p* < 0.05 are identified. Color denotes the *p*-value, and size of the spot represents the number of genes.

After the removal of duplicates, the potential targets in the GZD were 373 in number. The compound–target network of the GZD is shown in Figure 2A. Meanwhile, selecting from the GeneCards database, 2,356 OA-related targets were detected (Supplementary Table S3). The 166 overlapping genes between the two aforementioned analyses (Figure 2B, Supplementary Table S4) were identified as the candidate targets for GZD treatment in OA. Quercetin, kaempferol, and beta-sitosterol were the top three hub active ingredients identified from the network by using the degree method in the plugin CytoHubba.

### 3.3 GO functional and KEGG pathway enrichment analyses

For the 166 overlapping genes listed above, we performed GO enrichment analyses. The highly enriched terms of the biological processes were located in the positive regulation of gene expression, positive regulation of transcription from the RNA polymerase II promoter, and inflammatory response. In terms of the cellular components, the genes were mainly involved in the extracellular space, extracellular region, and macromolecular complex. As for the molecular function, enzyme binding, identical protein binding, and protein binding were the main terms (Figure 2C).

The pathways that were substantially altered by the GZD during OA therapy were discovered using the KEGG pathway analysis (Figure 2D). As indicated in the supplement (Supplementary Table S5), the KEGG enrichment analysis with the 15 most enriched KEGG terms (*p* < 0.05) showed that the main three enriched signaling pathways were the AGE-RAGE signaling pathway, IL-17 signaling pathway, and TNF signaling pathway.

### 3.4 DEGs identified by RNA-seq and their pathway enrichment analyses

**FIGURE 3:**
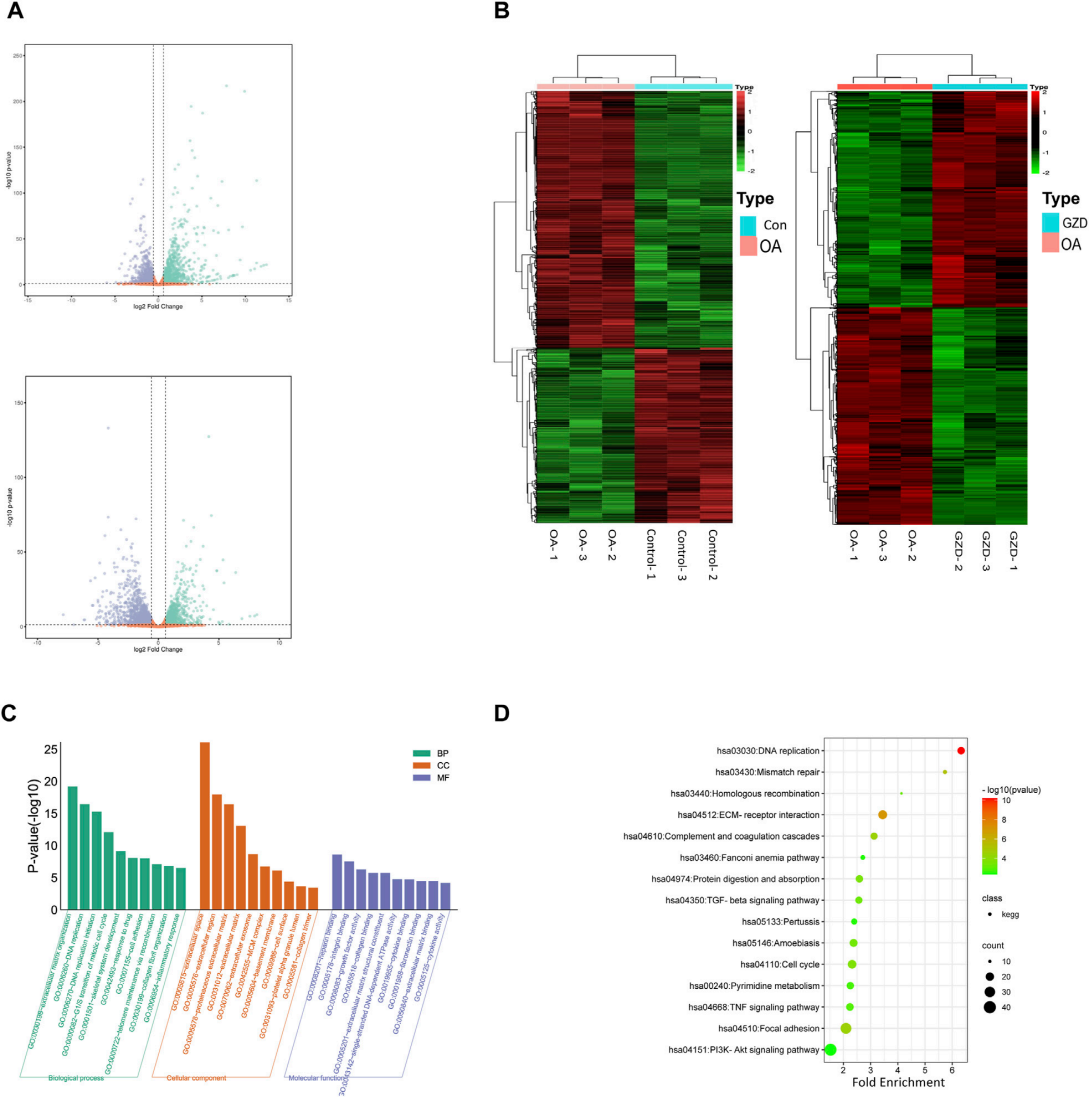
Differentially expressed genes between OA and GZD groups *via* RNA-seq. **(A)** Volcano map displays differential genes between the OA and Control (up) groups. Volcano map displays differential genes between the OA and GZD (below) groups. **(B)** Heatmap shows the scaled expression of differentially expressed genes defining the OA and Control (left) groups or the OA and GZD (right) groups, and red dots show that genes levels are upregulated. Green dots are downregulated. **(C)** Gene Ontology terms of candidate targets of GZD against OA. Top 30 GO functional categories with *p*-adjusted value <0.05 are selected. **(D)** KEGG pathway enrichment of candidate targets of GZD against OA. Pathways that had significant changes of *p* < 0.05 are identified.

The mRNA profiles of rat chondrocytes in both the OA and GZD groups were determined using mRNA-seq. In all, 22,250 genes were detected (Figure 3A). The thresholds for the DEGs were set as log |FC|≥1.0 and *p*-value < 0.05 with a FPKM value ≥0.1. A total of 546 upregulated and 880 downregulated DEGs (*p* < 0.05) were identified in the OA *vs.* GZD groups (Figure 3B, Supplementary Table S6). The analysis of the DEGs revealed a substantial difference mostly linked with extracellular space and heparin binding, according to the GO enrichment analysis (Figure 3C). The KEGG enrichment analysis with the 15 most enriched KEGG terms (*p* < 0.05) showed that the main three enriched signaling pathways were the TGF-beta signaling pathway, TNF signaling pathway, and PI3K-Akt signaling pathway (Figure 3D, Supplementary Table S7).

### 3.5 PPI network construction and identification of kernel targets

**FIGURE 4:**
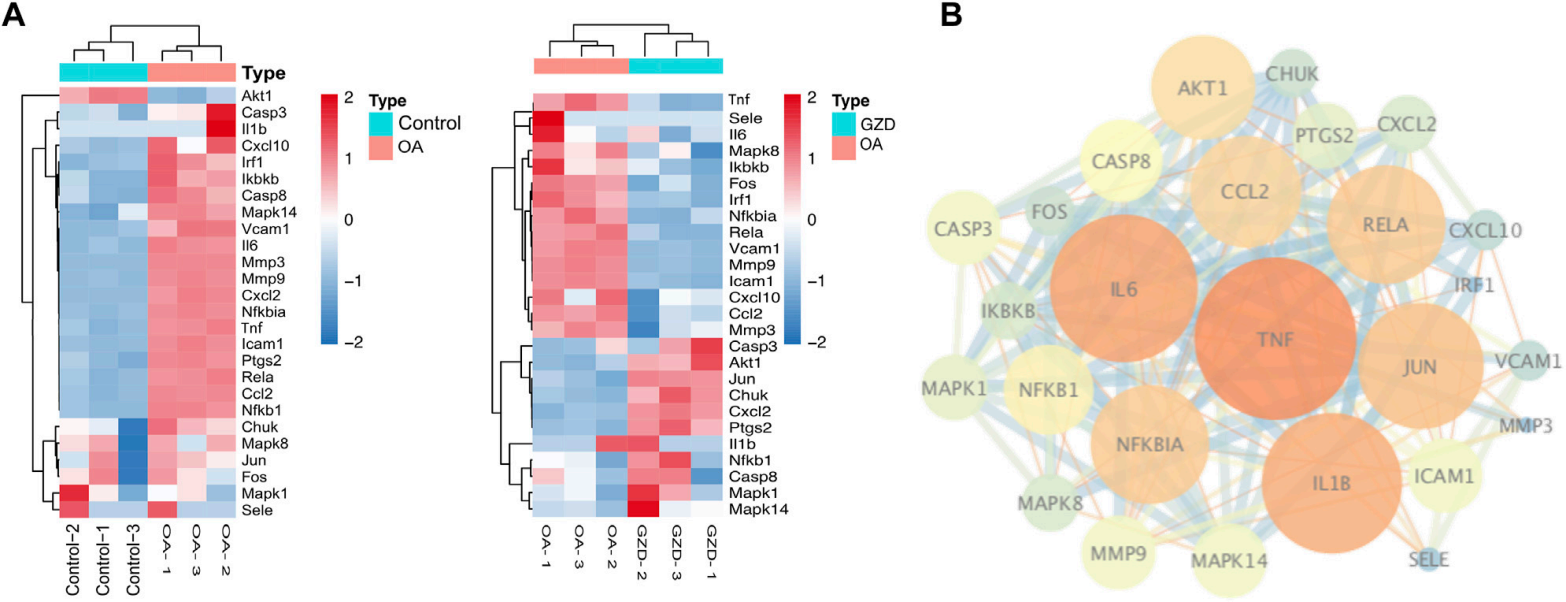
Analysis of hub genes in relation to RNA-seq of GZD-treated OA. **(A)** PPI network constructed genes in the key (TNF) signaling pathway; orange dots represent hub targets of the degree value in the OA and Control (left) groups or the OA and GZD (right) groups. **(B)** Heatmap shows the scaled expressions of differentially expressed genes defining the OA and GZD groups in the TNF signaling pathway, and red dots show that the gene level are upregulated and Green dots are downregulated.

According to the intersection of KEGG pathway enrichment analysis results of network pharmacology and RNA-seq, it can be demonstrated clearly that the TNF signaling pathway played a critical role in the effects of GZD treatment on OA. The DEG expressions in the TNF signaling pathway by RNA-seq were visualized using heatmaps in the three groups (Figure 4A). Then, 16 targets enriched in the TNF signaling pathway were used for building the PPI network (Figure 4B). By using the degree method in the plugin CytoHubba, the top three hub genes of TNF, IL-6, and IL-1β were identified from the network, which might be the kernel targets (Table 3).

**TABLE 3:** PPI network details sorted by the degree value from STRING interactions.

Gene name	Degree	Closeness centrality	Between centrality
TNF	24	0.96153846	0.120807
IL-6	22	0.89285714	0.0865267
IL-1β	21	0.86206897	0.06950806
JUN	19	0.80645161	0.04598966
RELA	18	0.78125	0.03898172
NFKBIA	18	0.78125	0.02738648
CCL2	17	0.75757576	0.04482275
AKT1	16	0.73529412	0.01779521
NFKB1	14	0.69444444	0.01368146
CASP8	13	0.67567568	0.00624351
ICAM1	12	0.65789474	0.00999868
MMP9	12	0.65789474	0.01065608
CASP3	12	0.65789474	0.00696453
MAPK14	12	0.65789474	0.00443795
PTGS2	11	0.64102564	0.00420106
MAPK1	11	0.64102564	0.00365091
CXCL2	10	0.625	0.00418519
MAPK8	10	0.625	0.00328728
IKBKB	10	0.625	6.08E-04
CHUK	9	0.6097561	2.38E-04
FOS	9	0.6097561	0.00283081
CXCL10	8	0.5952381	0.0045
VCAM1	8	0.5952381	0.00166667
SELE	6	0.56818182	0
MMP3	5	0.55555556	0
IRF1	5	0.55555556	0.00103175

### 3.6 GZD attenuates OA degradation

**FIGURE 5:**
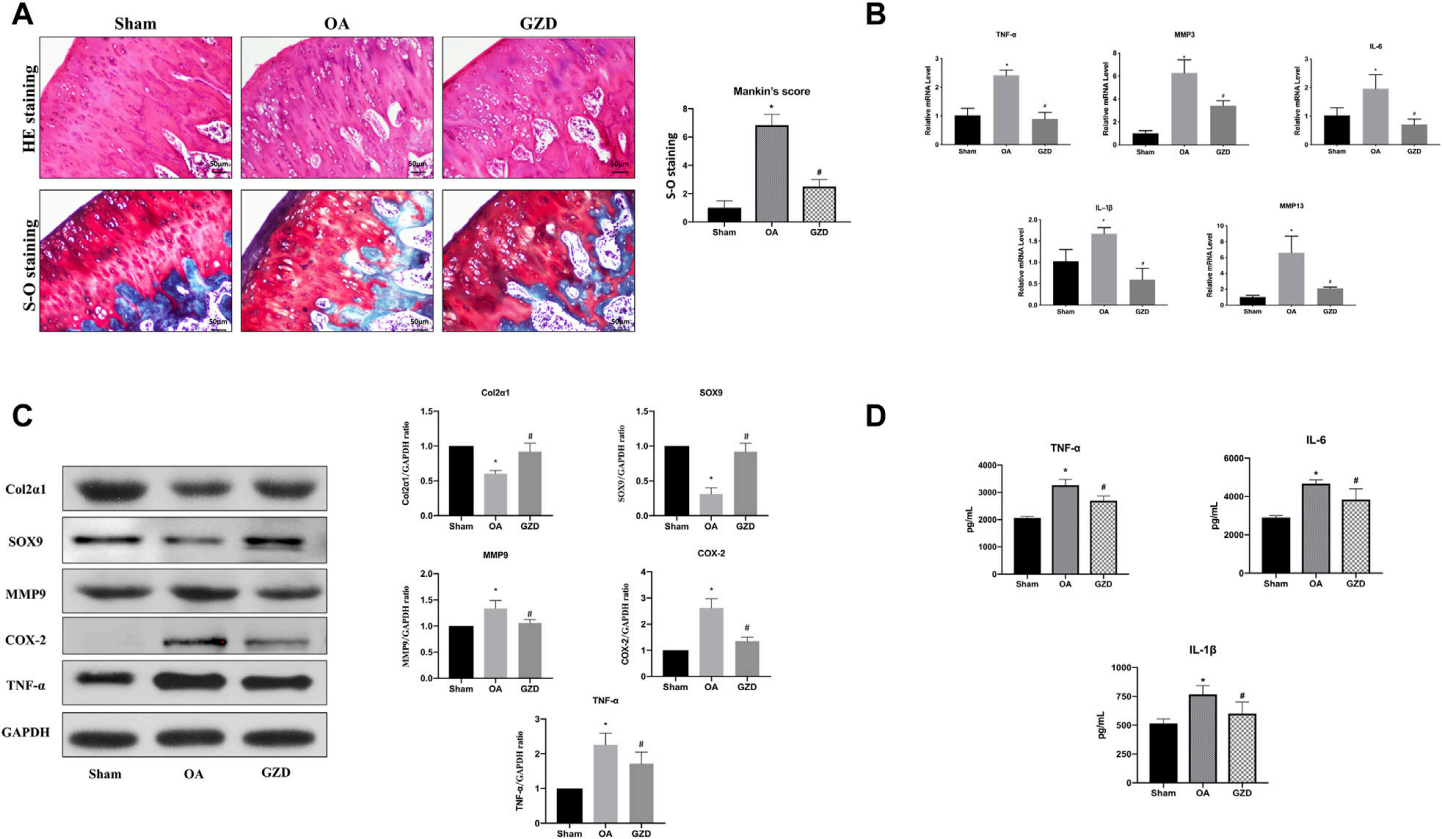
GZD ameliorated OA development *in vivo*. **(A)** Representative S-O staining and H&E staining of cartilage from the different experimental groups at 4 weeks post-surgery (scale bar = 50 µm), and the diagram shows the Mankin scores of the cartilage. **(B)** Inflammatory gene expression assessed *via* qPCR, with GAPDH for normalization. **(C)** Representative WB of the TNF signaling pathways and gene levels normalized to GAPDH expression *in vivo*. **(D)** Levels of TNF-α, IL-6, and IL-1β in the serum. Data are mean ± SD from three independent experiments. *p < 0.05 *vs.* Sham group; #*p* < 0.05 *vs.* OA group.

We then investigated the role of GZD in the treatment of OA *in vivo*. The representative staining of H&E and S-O for each group is illustrated after dehydration in Figure 5A. The sham group shows smooth cartilage surfaces and conserved S-O staining in the knee joint, while the OA group presents typical injury changes with a higher Mankin score (*p* < 0.05), such as massive hypocellularity, cartilage cauterization, and huge proteoglycan degradation. GZD group cartilage demonstrates a more positive red staining and smoother cartilage surface with a lower Mankin score (*vs.* OA group; *p* < 0.05).

### 3.7 GZD modulating TNF signaling *in vivo*

To further find the underlying mechanism behind the protective benefits of GZD therapy, we next analyzed the expression of transcription proteins involved in the TNF signaling pathways *via* qPCR and WB. The TNF-α, IL-1β, IL-6, MMP3, and MMP9 mRNA levels were found to be expressed at lower levels in the cartilage from the GZD group than in those from the OA group (*p* < 0.05) (Figure 5B). The proteins levels of both Col2α1 and SOX9 were higher in the GZD group than in the OA group (*p* < 0.05). The levels of MMP9, COX-2, and TNF-α in the GZD group were significantly lower than those in the OA group (*p* < 0.05) (Figure 5C).

### 3.8 GZD modulating serum levels of inflammatory markers

In addition, the serum levels of TNF-α, IL-6, and IL-1β which were the aforementioned hub genes identified through the PPI analysis were significantly higher in the OA group than in the sham operation group (*p* < 0.05), while the levels of these inflammatory markers in the GZD group were lower than those in the OA group (*p* < 0.05) (Figure 5D).

### 3.9 GZD modulating the TNF signaling *in vitro*

**FIGURE 6:**
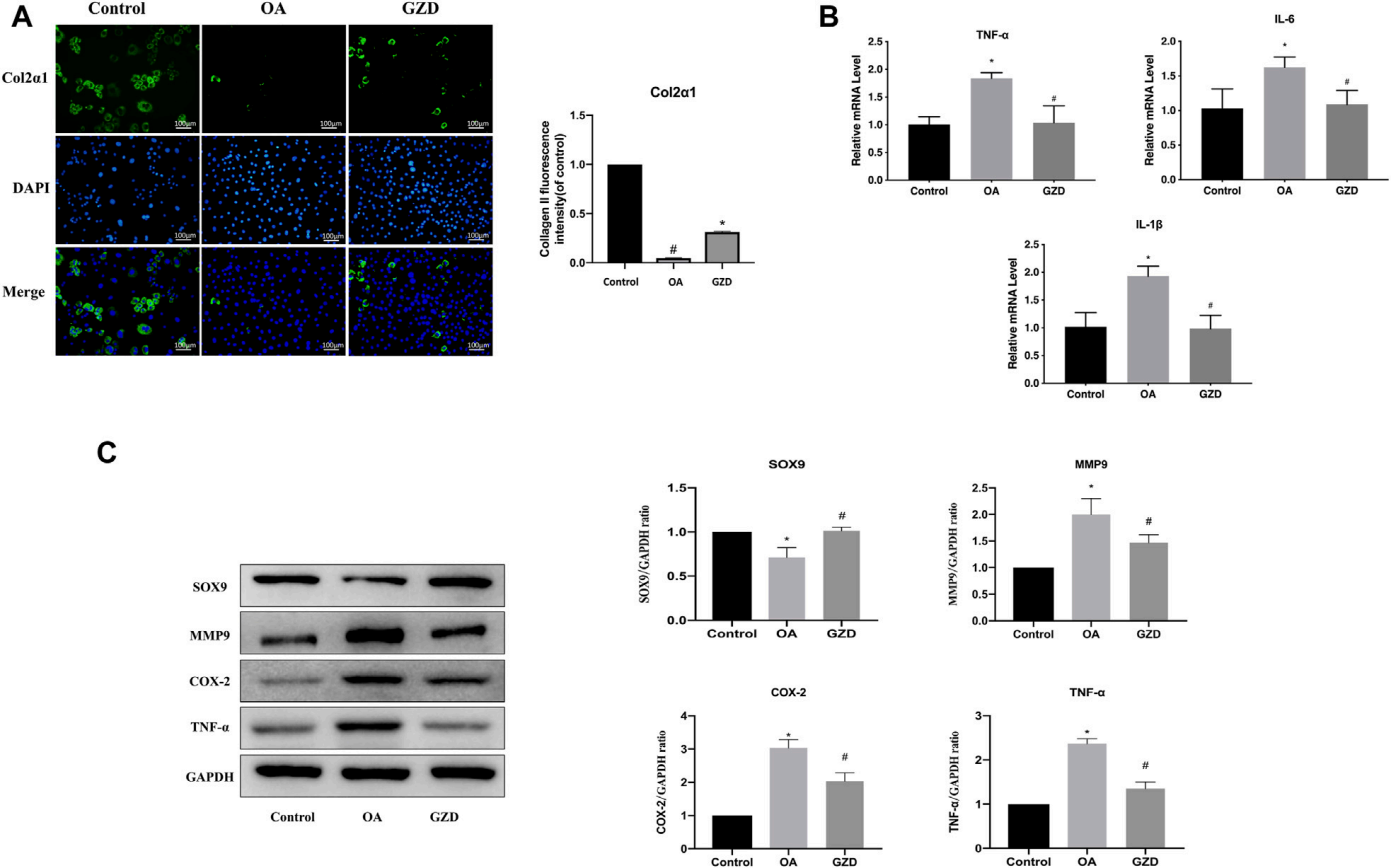
Analysis of the TNF signaling pathway changes in chondrocytes. **(A)** Representative fluorescent images of cells stained for Col2α1 (green), with DAPI being used for nuclear counterstaining (blue). Col2α1 fluorescent signal in these cells are quantified (right). **(B)** Expressions of hub genes (TNF-α, IL-6, and IL-1β) are assessed *via* qPCR, with GAPDH for normalization. **(C)** Representative WB of TNF signaling pathway and gene levels normalized to GAPDH expression *in vitro*. Data are mean ± SD from three independent experiments. *p < 0.05 *vs.* Control group; #*p* < 0.05 *vs.* OA group.

To clarify the TNF signaling pathway changes in chondrocytes, immunofluorescent staining, WB, and qPCR were assessed following the GZD treatment *in vitro*. The immunofluorescent staining analysis revealed that the Col2α1 protein level was significantly higher in the chondrocytes from the GZD group than in those from the OA group (Figure 6A). The transcriptome levels of TNF-α, IL-6, and IL-1β were lower in the GZD group than in the OA group (*p* < 0.05) (Figure 6B). The level of SOX9 in the GZD group was significantly higher than that in the OA group, while the expressions of MMP9, COX-2, and TNF-α were lower (*p* < 0.05) (Figure 6C).

## 4 Discussion

In light of the fact that OA is a highly prevalent and difficult-to-cure disease, it is urgent to find effective treatments to ameliorate its symptoms. In this study, we confirmed that GZD could decrease inflammatory cytokines and ameliorate cartilage degradation in *in vivo* experiments. The major active ingredients (quercetin, kaempferol, and beta-sitosterol) and hub genes (TNF, IL-6, and IL-1β) were identified. Their potential mechanisms were associated with the regulation of the TNF signaling pathway and inhibition of inflammatory response.

In the perspective of TCM, exploring the mechanism of GZD is particularly vital because GZD is not only regarded as the ancestor of classical formulas but also as the basis for herbal compounds treating syndromes of OA in later ages. The GZD is a Chinese herbal medicine composed of five herbs. Previous research on partial components of the GZD (two herbs of Paeoniae radix alba and licorice) strongly suggested that they may be valuable in treating and preventing OA through the analysis of network pharmacology (Zhu et al., 2019). However, there is a paucity of experimental data in supporting this hypothesis, leaving the process of whether GZD modified cartilage homeostasis still unclear.

The results of our compound–target network suggests that the ingredients of quercetin, kaempferol, and beta-sitosterol in GZD regulates multiple effects in OA treatment. These findings have also been corroborated by other independent studies. Quercetin, along with some of its conjugates, was approved for human use by the FDA (Magar and Sohng, 2020). They have various biological activities, such as changing cell cycle progression, encouraging cell proliferation, inhibiting apoptosis, and increasing autophagy (Magar and Sohng, 2020). According to Kanzaki et al., in a survey performed as a randomized, double-blind, placebo-controlled study, quercetin decreased the intensity of knee OA-associated symptoms better than the placebo (Kanzaki et al., 2012). As shown by Jiang et al. (2019), kaempferol had the ability to inhibit lipopolysaccharide-induced cell apoptosis, release pro-inflammatory cytokines by inhibiting miR-146a expression, and enhance the activity of the PI3K/AKT/mTOR signaling pathway in ATDC5. Kaempferol additionally could restrain the aggravation and extracellular grid debasement by balancing the XIST/miR-130a/STAT3 axis in C28/I2 cells (Xiao et al., 2021). In addition, as a widely distributed phytosterol with high oral bioavailability, beta-sitosterol exhibited the best anti-inflammatory effects, such as antipyretic, angiogenic, antihyperlipidemic, antiatherosclerotic, and antiarthritic effects (Xiao et al., 2021). Therefore, these ingredients may be representative compounds of GZD.

GZD exhibited ameliorating effects on OA, reducing joint damage such as in exaggerated inflammatory response and cartilage destruction, and pathological and serum cytokines *in vivo*; however, the underlying mechanisms by which GZD alleviates OA requires further clarification. Meanwhile, by analyzing the KEGG terms between network pharmacology and RNA-seq analysis, we identified that the TNF signaling pathway was one of the potential targets for GZD in the treatment of OA. TNF, which is generated by macrophages and chondrocytes, has been associated to synovitis and implicated in the degradation of the cartilage matrix. Various drugs interacting with TNF in influencing OA have been extensively reported (Yuan et al., 2010). The TNF signaling pathway has been linked to OA chondrocyte inflammation, NO production, and PGE2 release (Qin et al., 2012).

Then, we conducted a PPI network analysis to detect hub genes in the treatment of GZD. TNF, IL-6, and IL-1β were identified as the top three hub genes in the TNF signaling pathway. Clinical studies have also found that these inflammation markers are involved in the progression of OA signs and symptoms (Li et al., 2018). Another study on hip OA reported that positive associations between body composition and hip joint space narrowing were also mediated by IL-6, particularly in women (Stannus et al., 2010). As a verification, we further detected mRNA and proteins levels *via* qPCR and WB and found that their expressions were decreased under GZD intervention. In addition, the expression levels of anabolic proteins (Col2α1 and SOX9) were all higher in the GZD group than in the OA group, while the expression levels of the catabolic proteins (MMP9 and COX-2) showed the opposite tendency. Our results reflect the regulatory role of GZD on the key genes in the TNF signaling pathway *in vivo*. In OA healing, this signaling pathway is linked to cartilage development and angiogenesis, which may be involved in OA pathophysiology at a local level. Our results also reflect the changes of cartilage degeneration and systemic inflammation after GZD treatment.

Generally, TCM through the way of multi-molecular, multi-target, and multiple pathways acts on diseases achieving certain therapeutic effects. Our findings for network pharmacology and RNA-seq provide the same information that the regulatory function of GZD is a complex process involving multiple pathways, such as the TGF-beta signaling pathway, TNF signaling pathway, and PI3K-Akt signaling pathway. On the other hand, some limitations should be considered for a cautious interpretation. First, chondrocytes in RNA-seq were induced by IL-1β, and the results of genetic analysis may lead to inflammation. Second, network pharmacology has certain shortcomings in distinguishing the inhibition or activation effects as reported by Wu et al. (2019). Third, the hub gene and signaling pathway identified are only from network pharmacology prediction and the RNA-seq analysis. As the pathogenesis of OA is complex, the pathways mentioned above need to be further studied in the future. Moreover, our RNA-seq was detected only on single time point. Although the transcriptome can identify the molecular basis of many biological processes and various diseases, it cannot reflect the full complexity of biological functions.

In summary, through integrating network pharmacology and high-throughput RNA-seq, then further validating by *in vivo* and *in vitro* experiments, our present study has provided more precise and predictable molecular mechanism for GZD in treating OA.

## 5 Conclusion

In this study, the mechanism of GZD against OA was investigated by combining network pharmacological analysis, RNA-seq, and experimental verification. Its mechanism was related to the downregulation of the TNF signaling pathway and inhibition of OA inflammation.

## Data Availability

The data sets presented in this study can be found in online repositories. The names of the repository/repositories and accession number(s) can be found in the article/Supplementary Material.
