# Supplementary figures and images for: Molecular mechanisms of the Guizhi decoction on osteoarthritis based on an integrated network pharmacology and RNA sequencing approach with experimental validation
Source: Front Genet. 2023 Jan 25;14:1079631. doi: 10.3389/fgene.2023.1079631 (PMC9905689; doi:10.3389/fgene.2023.1079631)

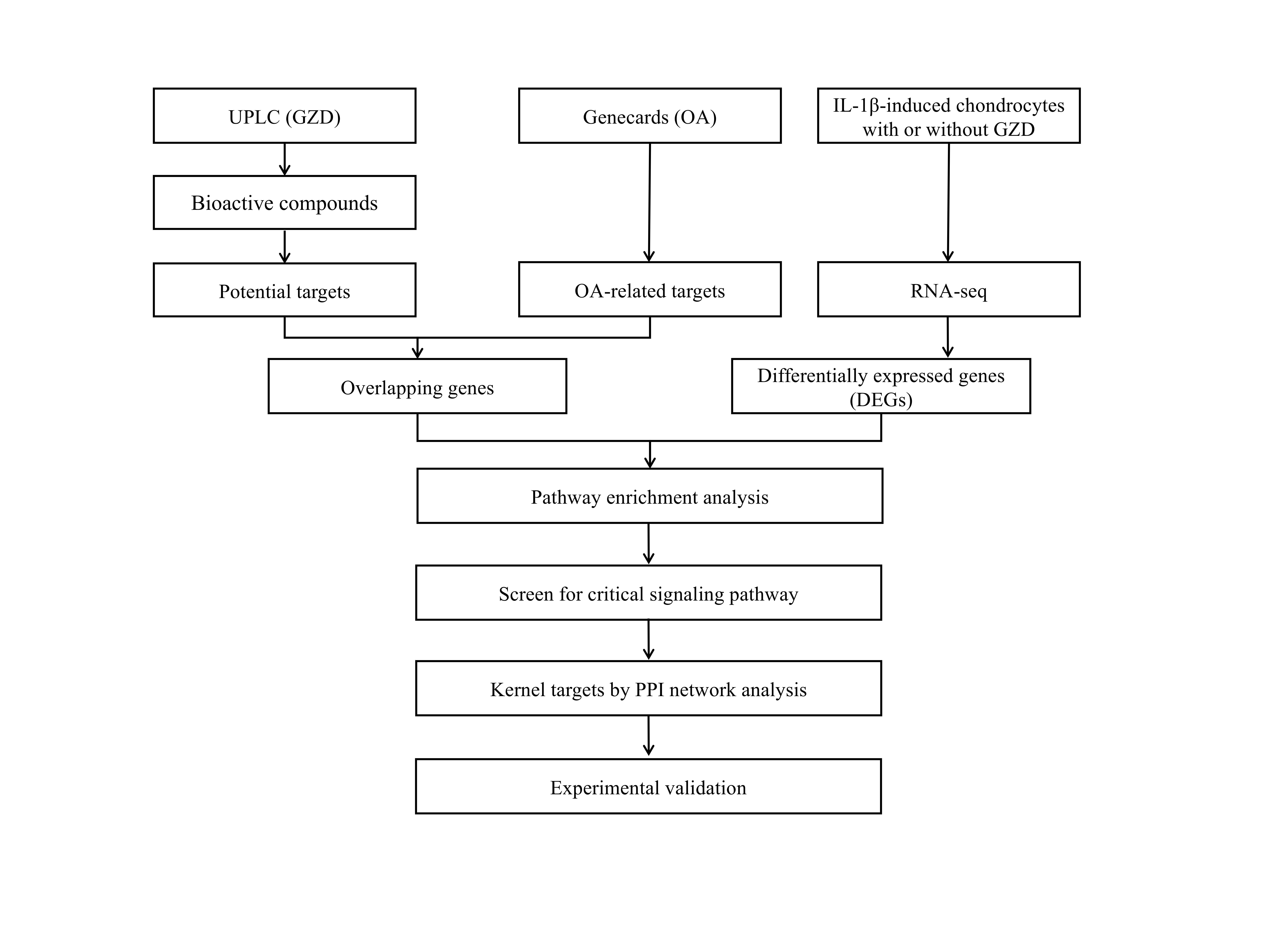

Supplement: Supplementary file 1 [file Image1.JPEG]

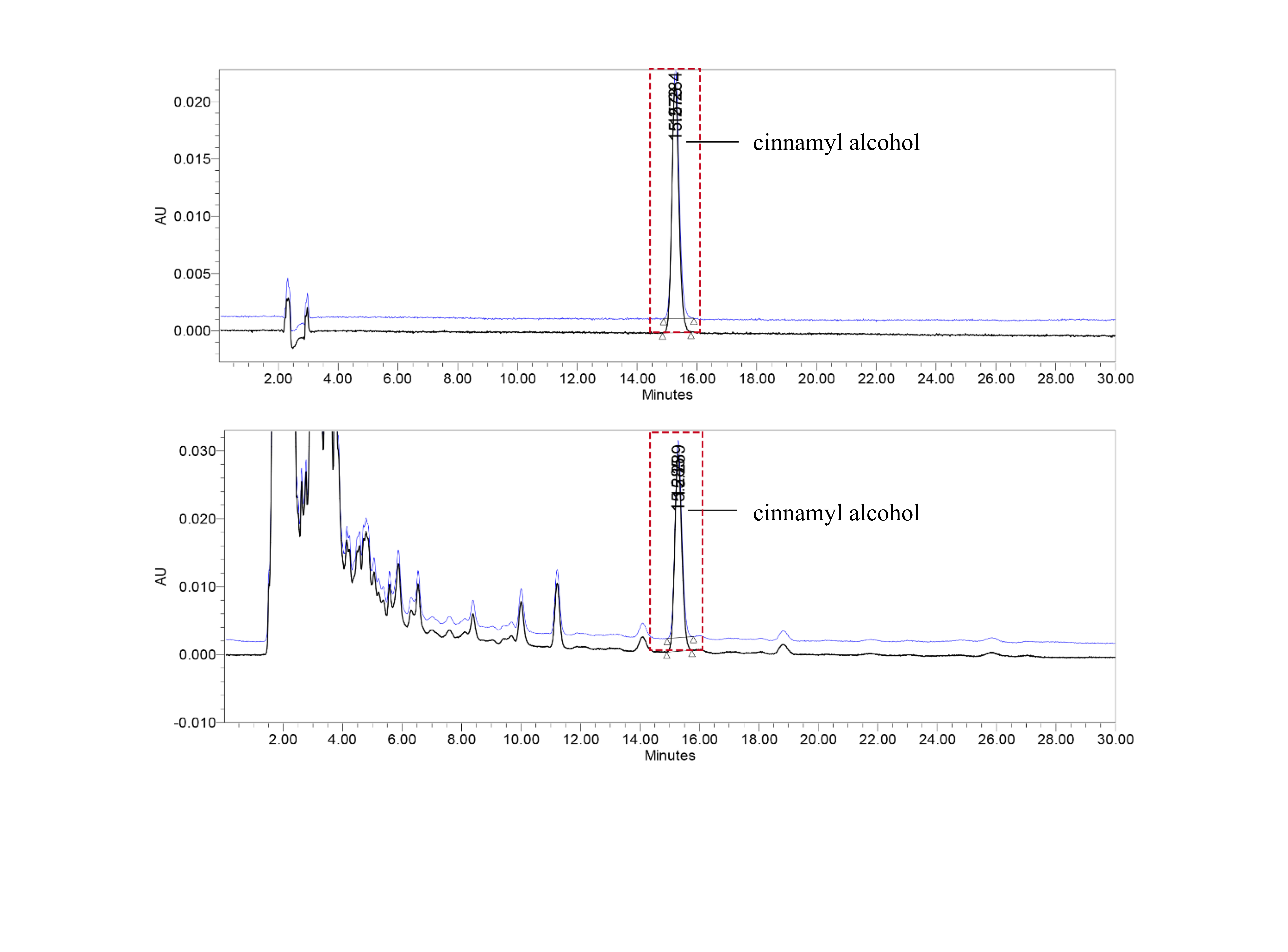

Supplement: Supplementary file 2 [file Image2.JPEG]
